# Upregulation of p16, Bax and Bcl-2 mRNA Expression Associated with Epithelial Apoptosis and Myofibroblast Proliferation in Kidney Fibrosis Model in Mice

**DOI:** 10.21315/mjms2020.27.2.4

**Published:** 2020-04-30

**Authors:** Ike Sulistiyowati, Junaedy Yunus, Dwi Cahyani Ratna Sari, Nur Arfian

**Affiliations:** 1 Department of Anatomy, Faculty of Medicine, Public Health and Nursing, Universitas Gadjah Mada, Yogyakarta, Indonesia; 2 Master Program in Biomedical Sciences, Faculty of Medicine, Public Health and Nursing, Universitas Gadjah Mada, Yogyakarta, Indonesia; 3 Department of Anatomy, Universitas Bengkulu, Indonesia

**Keywords:** UUO, kidney fibrosis, proliferation, cellular senescence, apoptosis, Bcl-2, p16, Bax

## Abstract

**Background:**

Cellular senescence may play a role in the development of kidney fibrosis, but its specific association with apoptosis or proliferation have yet to be determined.

**Objectives:**

This study aims to determine the effects of unilateral ureteral obstruction (UUO) on proliferation, cellular senescence and apoptosis in kidney fibrosis.

**Methods:**

A unilateral ureteral obstruction (UUO) procedure was performed to induce kidney fibrosis in 24 Swiss mice (3 months old, 30 g–40 g). Mice were sacrificed on day 3 (UUO3, *n* = 6), day 7 (UUO7, *n* = 6) and day 14 (UUO14, *n* = 6). Sham operation (SO) procedures were performed on the control group. The expression of Bcl-2, p16 and Bax mRNA was quantified with reverse transcription polymerase chain reaction (RT-PCR). Immunohistochemical (IHC) staining with anti-Bcl-2 and p53 antibodies was used to determine the localisation of proliferation and apoptosis. Data were analysed using one-way ANOVA followed by a post hoc least significant difference (LSD) test (*P* < 0.05)

**Results:**

RT-PCR analysis showed higher mRNA expression of Bcl-2, p16 and Bax in the UUO groups compared with SO group (*P* < 0.05). Immunostaining showed that Bcl-2 and p53 expression in tubular epithelium in the UUO groups, except Bcl-2 expression was found in interstitial areas of UUO14 group.

**Conclusion:**

Senescence in UUO might be associated with epithelial apoptosis and myofibroblast proliferation.

## Introduction

Chronic kidney disease (CKD) is a worldwide public health issue that affects millions of people from all racial and ethnic backgrounds and its prevalence is increasing every year (1). Risk factors of CKD include hypertension, diabetes, obesity and primary kidney disorders (2). These factors increase intraglomerular pressure, barrier filtration permeability, endothelial dysfunction, mesangial cell activation, podocyte and tubular cell counts and extracellular matrix synthesis, which collectively lead to kidney fibrosis (3).

Kidney fibrosis is a clinical endpoint of every chronic progressive kidney disease and is characterised by an excessive accumulation of the extracellular matrix (4). Kidney fibrosis is closely related to tubular injury, which can induce proliferation, autophagy, cellular senescence, epithelial-mesenchymal transitions (EMT) and apoptosis (5). Epithelial, fibroblast and myofibroblast cell proliferation are also part of the cellular processes driving kidney fibrosis (5) as well as apoptosis, which occurs in damaged tubular cells (6). The balance between anti-apoptosis proteins (Bcl-2) and proapoptosis proteins (Bax) determines whether the tubular cells will survive and proliferate or undergo apoptosis (7). Cellular senescence can also be triggered by repeated CKD-related stress exposures (8) and aging, DNA damage, oncogene activation, oxidative stress and mechanical stress (9). The p16 protein is a cellular senescence marker that inhibits cyclin-dependent kinase 4 (CDK4) and cyclin-dependent kinase 6 (CDK6) (10).

Unilateral ureteral obstruction (UUO) is a widely used experimental model of renal injury that is performed by unilaterally ligating and cutting a ureter (11). There is a decrease in renal blood flow and glomerular filtration rate (GFR) during the first 3 h after UUO, followed by a progressive increase in interstitial inflammatory cell inflltration occurring 12 h to 14 days after the obstruction and by day 3, inflammation, microvascular damage and increased numbers of fibroblasts are apparent (12). Interstitial fibrosis occurs between days 5 and 12 and reaches its peak on day 19 (13).

Several studies have been published on proliferation, cellular senescence and apoptosis using the UUO mouse model. However, none of these studies have comprehensively reported on the progression of UUO from its induction to its pathophysiological endpoint. To address this, we studied the effects of UUO on cellular proliferation, senescence, and apoptosis in the context of kidney fibrosis. Understanding the effects of UUO on cellular proliferation, aging and apoptosis in the context of renal fibrosis from its early to advanced stages could facilitate the development of novel treatments for kidney fibrosis.

## Methods

This was a quasi-experimental study with a that consisted of control and treatment groups.

### The Unilateral Ureteral Obstruction Model

Three-month-old male Swiss mice weighing 30 g–40 g were used as the UUO (*n* = 18) and SO (*n* = 6) subjects. The mice were anesthetised using a 0.01 mg/g body weight (BW) cocktail solution (ketamine 60 mg, xylazine 10 mg, acepromazine 2 mg) via intraperitoneal injection. The right flank region of each mouse was opened and the proximal and distal right ureter was ligated with silk 0.4 before an incision was made between them. The mice were sacrificed on days 3, 7 and 14 for the UUO3, UUO7 and UUO14 groups, respectively. The SO were performed by conducting a laparotomy without performing the ureteral ligation.

### RNA Extractions and cDNA Synthesis

Total RNA was extracted from 50 mg–100 mg kidney pieces using Genezol (Geneaid GZR100, Geneaid Biotech Ltd, New Taipei City, Taiwan) and quantified using a Nanodrop. The amount of 3,000 total RNA was added for cDNA conversions with Rever Tra Ace^®^ (Toyobo Cat. No. TRT-101, Osaka, Japan) and random primers (Toyobo Cat. No. 3801) with the following polymerase chain reaction (PCR) conditions: 30 °C for 10 min (denaturation), 42 °C for 60 min (annealing) and 99 °C for 5 min (extension).

### Reverse Transcriptase PCR and Electrophoresis

Reverse transcription PCR (RT-PCR) was used to amplify the following cDNAs: Bcl-2 *(reverse:* GCA TCC CAG CCT CCG TTA TCA and *forward:* ACC CTG TTG TGT AGC CGT CTG), p16 (*reverse*: CTC GCA GTT CGA ATC TGC AC and *forward*: TGC AGA TAG ACT AGC CAG GC), Bax (*reverse*: GCC TTG AGC ACC AGT TTG CT and *forward*: GCT TAC CGT AGC AGT TGG AT), and glyceraldehyde 3-phosphate dehydrogenase (GAPDH) (*reverse*: TCT CGC TCC TGG AAG ATG GT and *forward*: GGC ACA GTC AAG GCT GAG AT), which was used for normalisation.

Reverse Transcriptase PCR was prepared with a master mix of 3 μL cDNA, 12.5 μL of Taq master mix (GoTaq®Green Master Mix, Cat. No. M7128), 0.6 μL of forward and reverse primers and 8.3 μL of PCR water. The cDNA were amplified using the following programme: 94 °C for 2 s (initial denaturation), 94 °C for 10 s (denaturation), 20 s at 57 °C, 51 °C, 55 °C or 57 °C for Bcl-2, p16, Bax or GAPDH, respectively (annealing), 72 °C for 1 min (extension) and 72 °C for 10 min (final extension) for a total of 35 cycles. The PCR products were analysed using 2% agarose gel with a 100 bp DNA ladder (Bioron Cat. No. 306009, Germany). Gene expression was quantified by densitometry analysis using ImageJ software with GAPDH as a housekeeping gene.

### Histopathological Examination

The kidneys were embedded in paraffin blocks and 4 μm sections were stained with periodic acid-Schiff (PAS). The stained sections were examined using an Olympus CX22 (Olympus Corporation, Tokyo, Japan) light microscope, and the images were captured using OptiLab software at 400× magnification to visualise the tubular injuries.

### Immunohistochemical (IHC) Staining

The kidneys were embedded in paraffin blocks cut into 4 μm sections, deparaffinised, heated in a citrate buffer at pH 6 for antigen retrieval and blocked against endogenous peroxidase using 3% H_2_0_2_ in a PBS solution. The slides were incubated with background sniper (Biocare Medical STUHRP700, CA, USA) followed by the primary antibody incubation (rabbit primary monoclonal antibody Bcl-2 at a 1:200 dilution (Bioss bs-0032R, Massachusetts, USA) and rabbit primary monoclonal antibody p53 at a 1:100 dilution (Abcam ab131442, Cambridge, UK)) overnight at 4 °C. The slides were then incubated with the appropriate secondary antibody (Trekkie Universal Link-Biocare Medical STUHRP700, CA, USA), TrekAvidin-HRP and diaminobenzidine tetrahydrochloride (DAB) (Biocare, STUHRP700H L10). The slides were visualised on a light microscope (Olympus CX22), analysed using ImageJ software, and portrayed using OptiLab software at 400× magnification.

### Statistical Analysis

The data were analysed using SPSS software (IBM SPSS statistics version 25.Ink) and Shapiro-Wilk tests for distribution analysis. Since the data were normally distributed, multiple comparisons among the groups were conducted by one-way ANOVA followed by post hoc LSD tests. A threshold of *P* < 0.05 was defined as statistically significant.

## Results

### Tubular Injury

**Figure 1:**
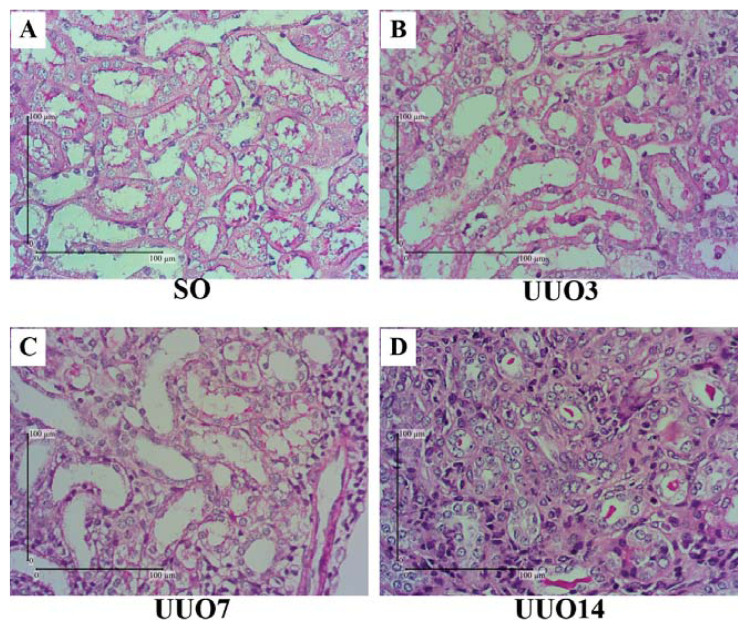
Renal histology in the UUO model with PAS staining. (A) The SO group: the tubular lumen is not dilated, the tubular basal membrane is intact, there is a brush border. (B, C) UUO3 and UUO7 groups: dilated tubules, contracted tubular basement membranes, dilated interstitial space, inflammatory cell accumulation, loss of brush border and the presence of intraluminal casts. (D) UUO14 group: atrophic tubules, very wide interstitial space, fibrotic cell accumulation and inflammatory cells in the interstitial space

When compared with the SO group, the UUO groups generally presented with tubular injuries that were characterised by a loss of brush border, tubular atrophy, tubular dilatation and the presence of intraluminal casts (Figure 1A). The UUO3 group experienced tubular dilatation, shrinkage of the tubular basement membrane, widening of the interstitial space due to the accumulation of inflammatory cells, a loss of brush border and the presence of intraluminal casts (Figure 1B). The severity of these features increased in the UUO7 group (Figure 1C) and the UUO14 group presented with chaotic histological features that included atrophic tubules and very wide interstitial spaces that contained an accumulation of fibrotic and inflammatory cells (Figure 1D).

### Proliferation Activities During UUO

**Figure 2:**
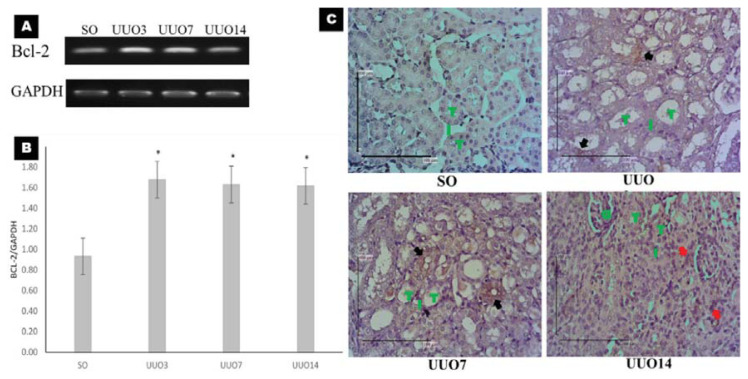
The mRNA and protein expression of Bcl-2 as a proliferation marker. (A) Gel electrophoresis figures of the RT-PCR analyses of Bcl-2 and GAPDH from kidney tissue. (B) Bar charts showing relative quantification of the mean mRNA expression of Bcl-2/GAPDH. The data were analysed by one-way ANOVA and LSD tests. Asterisks show significant differences between SO and UUO groups (*P < 0.05). (C) IHC for Bcl-2 to visualise the localisation of proliferating cells. Bcl-2 protein expression was found in the tubular epithelial cells (black arrows) in the UUO3 and UUO7 groups and in the interstitial space (red arrows) of the UUO14 group. Scale bar = 100 μm

There was increased Bcl-2 mRNA expression in the UUO3 (1.68 [0.23]; *P* = 0.001), UUO7 (1.63 [0.46]; *P* = 0.002) and UUO14 (1.62 [0.22]; *P* = 0.003) groups compared with that in the SO group (0.93 [0.22]), which indicated increased cellular proliferation in the UUO groups relative to that in the SO group. Bcl-2 mRNA expression was highest in the UUO3 group (1.68 [0.23]) and decreased in the UUO7 (1.63 [0.46]) and UUO14 (1.62 [0.22]) groups (Figures 2A and 2B). However, there was no significance difference among UUO groups. IHC using anti-Bcl-2 antibodies was used to localise proliferating cells. Protein expression of Bcl-2 was found in the tubular epithelial cells of the UUO3 and UUO7 groups and in the interstitial areas of the UUO14 group (Figure 2C).

### Cellular Senescence During UUO

**Figure 3:**
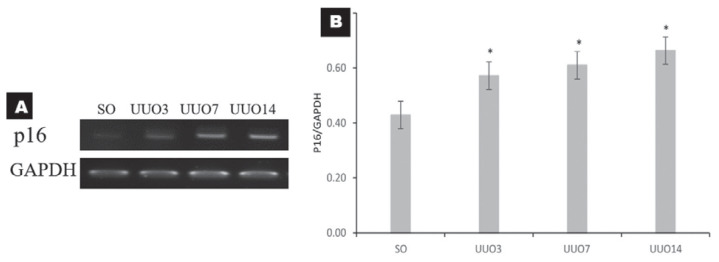
The mRNA expression of p16 as a marker of cellular senescence. (A) Gel electrophoresis figures of the RT-PCR analyses of p16 and GAPDH from kidney tissue. (B) Bar charts showing relative quantification of the mean mRNA expression of p16/GAPDH. The data were analysed by one-way ANOVA. Asterisks show significant differences between SO and UUO groups (*P < 0.05)

There was increased p16 mRNA expression in the UUO3 (0.57 [*P* = 0.018], UUO7 (0.61 [0.10]; *P* = 0.003) and UUO14 (0.66 [0.10]; *P* = 0.001) groups compared with that in the SO group (0.43 [0.08]), which indicated increased cellular senescence activity in the UUO groups relative to that in the SO group. The mRNA expression of p-16 was lowest in the UUO3 (0.57 [0.06]) group and elevated in the UUO7 (0.61 [0.10]) and UUO14 (0.66 [0.10]) groups (Figures 3A and 3B). RT-PCR analysis also showed consistently higher expression in UUO groups, however it revealed no significance difference among UUO groups.

### Apoptosis Activities During UUO

**Figure 4:**
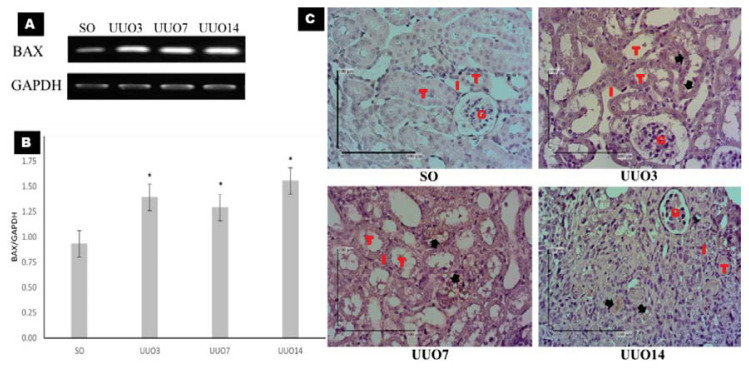
The mRNA expression of Bax and protein expression of p53 as markers of apoptosis. (A) Gel electrophoresis figures of the RT-PCR analyses of Bax and GAPDH from kidney tissue. (B) Bar charts showing the relative quantification of the mean mRNA expression of Bax/GAPDH. The data were analysed by one-way ANOVA test. Asterisks show significant differences between the SO and the unilateral ureteral obstruction (UUO) groups (*P < 0.05). (C) IHC of p53 to visualise the localisation of apoptotic cells. Protein expression of was found in the tubular epithelial cells (black arrows) of the UUO3, UUO7 and UUO14. Scale bar = 100 μm

There was increased mRNA expression of Bax in the UUO3 (1.39 [0.24]; *P* = 0.002), UUO7 (1.29 [0.25]; *P* = 0.012) and UUO14 (1.55 [0.18]; *P* = 0.000) groups compared with that in the SO group (0.93 [0.21]), which indicated increased apoptotic activity in the UUO groups relative to that in the SO group. The mRNA expression of Bax was lowest in the UUO7 (1.29 [0.25]) group and highest in the UUO14 (1.55 [0.18]) group (Figures 4A and 4B). However, there were not significant difference among UUO groups. We used IHC with anti-p53 antibodies to identify apoptotic cells, which were localised to the renal tubular cells of the UUO3, UUO7 and UUO14 groups (Figure 4C).

## Discussion

The UUO is a popular experimental model of renal injury which causes subacute renal injury that is characterised by tubular cell injury, interstitial inflammation and fibrosis. Ligating the ureter causes tubular dilatation and injury to the tubular epithelial cells, which triggers an inflammatory response (12). Kidney damage initiates a repair response that can either be adaptive, leading to the restoration of a normal epithelium or maladaptive epithelium, which may lead to fibrosis and CKD. This finding is indicated by the elevated mRNA expression of Bcl-2 in the UUO groups compared with that in the SO group as well as the localisation of Bcl-2 protein expression (Figure 2). The adaptive repair response involves adaptive tubular epithelial cell proliferation (14). However, repeated injuries can cause maladaptive repair responses that are characterised by interstitial fibroblast and myofibroblast proliferation (15). These results are consistent with the research conducted by Zhang et al. (16) in mice that underwent UUO on the 3rd, 7th and 11th day. In this study, they used IHC staining with proliferating cell nuclear antigen (PCNA) antibodies to study tubulointerstitial proliferation, which was higher in the UUO groups than in the SO group. IHC staining with Bcl-2 antibodies also showed a significant increase in Bcl-2 in the tubular cells of the UUO groups compared with the SO group. These data indicated that there was increased proliferation in the UUO group relative to the SO group (16).

Repeated exposure to injuries can also lead to cellular senescence. While many of the tubular epithelial cells undergo apoptosis, some will transition into cellular senescence (17). In UUO, injuries are continuous and increasingly widespread, which increases the amount of cellular senescence (as shown in Figure 3) and p16 mRNA expression in the UUO groups compared with those in the SO group. The number of senescent cells increases due to their inefficient elimination by the body’s immune system and their ability to secrete senescence-associated secretory phenotype (SASP), which can induce other surrounding cells to become senescent (18). These findings are consistent with research by Wolstein et al. (19) who reviewed the effects of p16 as a marker of cellular aging in renal fibrosis by comparing normal mice with a INK4a KO model using UUO and sacrificing on days 1, 3 and 7. They also measured p16 expression by real-time PCR. Their results showed increased p16 expression in normal mouse kidneys following UUO on days 3 and 7 compared with controls (19).

Apoptosis is the main mechanism of cell death in UUO (11). Ubiquitin-proteasome pathway (UUP)-related apoptosis can be induced by tissue hypoxia from disrupted blood circulation in the kidneys, inflammatory mediator infiltration and mechanical stress due to tubular dilatation (20). Tubular cell death is present one day after UUO and reaches its peak at two weeks due to the widespread processes involved in tissue injury (12). Our results were consistent with these observations with the highest levels of Bax expression occurring in the UUO14 group. However, the lowest levels of Bax expression were seen in the UUO7 group (Figure 4). This is likely due to self-protection mechanisms that are initiated by injured tubular cells. These cells can express both Bcl-2 and Bax, which are part of the self-protection mechanism. Injured cells with higher Bax expression will survive if there is a simultaneous increase in Bcl-2 or vice versa (16). These results are consistent with research conducted by Xu et al. (20), which used UUO mice at days 3, 7 and 14 to test for apoptosis with TUNEL staining. They also found significantly increased apoptotic activity in the proximal tubular cells of the UUO groups compared with that in the SO group (20).

The processes of apoptosis, cellular senescence, and apoptosis with kidney fibrosis can all influence each other. Tubular injury and kidney fibrosis can lead to complex pathological processes that increase oxidative stress, inflammation, autophagy, apoptosis, and cellular senescence (21). Senescent cells remain metabolically active and secrete factors that can change the surrounding cellular environment (8), including SASP, which consists of soluble and insoluble factors (9). These factors affect the surrounding cells by activating various cell surface receptors and their corresponding signal transduction pathways (8, 22).

Kidney injury activates tissue repair mechanisms in which SASP has positive initial effects by inducing pro-inflammatory cytokines and growth factor secretion to trigger proliferation (23). Simultaneously, injury-related inflammation increases the expression of IL-6, tumour necrosis factor-α (TNF-α) and monocyte chemotactic protein-1 (MCP-1), which recruit various immune cells to support tissue repair by removing damaged antigens and cells, maintaining homeostasis, and increasing SASP secretion (8). However, when injuries are chronic and the tissue cannot be repaired, the accumulation of SASP results in further tissue damage due to increased numbers of senescent cells, chronic inflammation and disrupted homeostasis (24).

## Conclusions

Our findings show significantly increased proliferative activity, cellular senescence and apoptosis in the UUO groups when compared with the SO group. Future studies should examine the impacts of cellular senescence in kidney fibrosis by using double-label immunofluorescence staining.
